# A Cationic Amphiphilic Random Copolymer with pH-Responsive Activity against Methicillin-Resistant *Staphylococcus aureus*

**DOI:** 10.1371/journal.pone.0169262

**Published:** 2017-01-06

**Authors:** Sungyoup Hong, Haruko Takahashi, Enrico T. Nadres, Hamid Mortazavian, Gregory A. Caputo, John G. Younger, Kenichi Kuroda

**Affiliations:** 1 Department of Emergency Medicine, College of Medicine, The Catholic University of Korea, Seoul, Republic of Korea; 2 Department of Biologic and Materials Sciences, University of Michigan School of Dentistry, Ann Arbor, Michigan, United States of America; 3 Department of Chemistry and Biochemistry, Rowan University, Glassboro, New Jersey, United States of America; 4 Department of Emergency Medicine, University of Michigan Medical School, Ann Arbor, Michigan, United States of America; Nanyang Technological University, SINGAPORE

## Abstract

In this report, we demonstrate the pH-dependent, *in vitro* antimicrobial activity of a cationic, amphiphilic random copolymer against clinical isolates of drug-resistant *Staphylococcus aureus*. The polymer was developed toward a long-term goal of potential utility in the treatment of skin infections. The proposed mechanism of action of the polymer is through selectively binding to bacterial membranes and subsequent disruption of the membrane structure/integrity, ultimately resulting in bacterial cell death. The polymer showed bactericidal activity against clinical isolates of methicillin-resistant or vancomycin-intermediate *S*. *aureus*. The polymer was effective in killing *S*. *aureus* at neutral pH, but inactive under acidic conditions (pH 5.5). The polymer did not exhibit any significant hemolytic activity against human red blood cells or display cytotoxicity to human dermal fibroblasts over a range of pH values (5.5–7.4). These results indicate that the polymer activity was selective against bacteria over human cells. Using this polymer, we propose a new potential strategy for treatment of skin infections using the pH-sensitive antimicrobial polymer agent that would selectively target infections at pH-neutral wound sites, but not the acidic, healthy skin.

## Introduction

Drug-resistant bacterial infections have been rapidly increasing over the last several decades, although resistance to synthetic antibiotics has been noted since their widespread application as early as 1940. Recently, healthcare- and community-associated *Staphylococcus aureus* have become a major concern to patients, with community-acquired infections becoming more common [1]. However, conventional antibiotics such as fluoroquinolones and daptomycin may no longer be viable options for treatment of bacterial infections in clinical situations due to increased resistance [2]. In these cases, vancomycin has been considered the antibiotic of last resort, but the increased frequency of reports of vancomycin intermediate *S*. *aureus* (VISA) and vancomycin resistant *S*. *aureus* (VRSA) suggest that drug resistance among *S*. *aureus* will continue to be a clinical challenge for the foreseeable future [3]. It has been a scientific challenge to develop new antimicrobial compounds which have a novel mechanism effective in inhibiting growth of drug-resistant bacteria [4–6].

The therapeutic potential of host-defense antimicrobial peptides (AMPs) found in the innate immune system has been explored as candidates for the development of new antimicrobials [7]. These molecules have been identified in a wide variety of organisms including insects, reptiles, and up through mammals [8]. Many AMPs have been shown to be active against drug-resistant bacteria and generally do not contribute to the resistance development in bacteria, likely due to differences in mechanism of action [7–10]. While there is no general consensus sequence among the evolutionarily diverse AMPs, generally they are relatively low molecular weight (10-50aa), and are often rich in cationic and hydrophobic residues resulting in an amphiphilic nature [9]. The cationic residues enhance the binding of these AMPs to anionic bacterial membranes. Because human cell membranes have significantly lower net negative charge, and this charge is localized to the cytosolic face of the membrane, electrostatic interactions result in AMPs preferentially binding to bacterial cell membranes, imparting inherent selectivity to bacteria over human cells. The proposed mechanism targets a fundamental cellular structure, the lipid membrane, which bacteria cannot “evolve” a resistance against, which is consistent with the presence of AMPs throughout the evolutionary tree [8]. While attractive in their novelty and low resistance potential, there are significant limitations for clinical use of AMPs [11]. Chief among them are high manufacturing cost, low stability due to proteolytic degradation, and low oral availability [11].

In an attempt to develop new antimicrobials which are effective against antibiotic resistant bacteria and address the issues described above, we previously designed and developed non-peptide cationic amphiphilic random copolymers consisting of cationic and hydrophobic side chains [12]. These synthetic copolymers were designed to mimic the mode of action of AMPs but not necessarily the helical secondary structures commonly found in amphiphilic AMPs. The selective antimicrobial activity of AMPs is directly linked to the cationic and hydrophobic amino acids in the peptide sequences, and thus these same functionalities were designed into the polymer structure. This synthetic polymer structure based in methacrylate was selected from a library of related structures for further study because of potent activity and cell selectivity [13]. Specifically, the cationic groups of polymer were incorporated to bind to enhance electrostatic interactions with anionic bacterial membranes, providing selective activity against bacteria. The hydrophobic groups were included to drive the insertion of polymer chains into bacterial membranes, causing membrane disruption. In our previous work, these polymers exhibited broad spectrum activity, rapid bactericidal activity, and low propensity for resistance development in bacteria, which are the hallmarks of the AMPs the polymers are designed to mimic [14].

*S*. *aureus* is a commonly encountered agent of skin infections, and prevention of community associated, drug-resistant *S*. *aureus* infections are lagging behind similar efforts in hospital settings [15]. In general, the pH values of normal and infected skin tissues are largely different; the normal skin surface is acidic due to the acid mantle, yielding a typical pH in the range 5.4–5.9 for human skin, although the reported pH values are varied depending in literature primarily due to different methods for measurement of skin pH [16]. The normal, acidic environment inhibits bacterial growth as well as suppresses the activity of proteases which are harmful to the tissue [16]. However, the pH of infected sites is close to neutral because of the exposure of subcutaneous tissue [17].

In this report, we investigated the *in vitro* antimicrobial activity of a cationic amphiphilic random copolymer against clinical isolates of drug-resistant *S*. *aureus*. We also focused on the pH-dependence of the antimicrobial activity of this polymer. These two areas are relevant to the long-term interest in the potential application of these polymers as a topical antimicrobial for the treatment of *S*. *aureus* skin infections. The goal of this work is to exploit the inherent pH differences in normal vs. infected skin which may impact the amphiphilic balance of the polymers as well as the cellular properties of the infecting bacteria, resulting in pH-sensitive susceptibility profiles. It is critical to know the pH-dependence on antimicrobial activity of the polymer to determine if the polymer could potentially be effective as a topical antibiotic agent toward treating skin infections. In this study, we characterized the *in vitro* antimicrobial activity against drug-resistant *S*. *aureus* as well as the cytotoxicity of the polymer to human cells. The results indicated that the polymer was active against clinical isolates of drug-resistant *S*. *aureus*. The results also indicated that the polymer was active under the neutral pH conditions similar to that of an infected site, but were inactive under acidic conditions similar to the normal pH of skin, furthering the potential for future application as a topical antimicrobial agent.

## Materials and Methods

### Materials

Twelve MRSA colonies (including three VISA colonies containing the mecA gene) which were isolated from the blood of ten patients treated for blood stream infections at the University of Michigan Health System were used (Table 1). Human adult fibroblasts (PCS-201-012) and fibroblast basal medium (ATCC PCS-201-030) were obtained from ATCC (Manassas, VA, USA). Muller-Hinton (MH) broth was purchased from Fisher Scientific (Pittsburg, PA, USA). Vancomycin hydrochloride was purchased from Hopira, Inc. (Chicago, IL, USA). 96-well plates were purchased from Corning (Constar 3591, Corning, NY, USA). Cell counting kit-8 (CCK-8) was purchased from Dojindo laboratories (Kumamoto, Japan). 4-Amino-1-butanol was purchased from TCI Chemicals. Di-*tert*-butyl dicarbonate was purchased from Oakwood Chemicals. 2,2-azobisisobutyronitrile (AIBN) was purchased from Sigma-Aldrich (St Louis, MO). 2-cyanoprop-2-yl-dithiobenzoate was purchased from Strem Chemicals (Newburyport, MA). Trifluoracetic acid (TFA) and the solvents hexanes, dichloromethane, diethyl ether and methanol were purchased from Fisher Scientific. Ethyl methacrylate (EMA), methacryloyl chloride, 4-butanolamine, methyl 3-mercaptopropionate and di-*tert*-butyldicarbonate were purchased from Acros Organics (part of Thermo Fisher Scientific, Geel, Belgium).

**Table 1:** Susceptibility of MRSA to vancomycin, mupirocin, and PE_31_.

Colony^a^	Vancomycin		Mupirocin		PE_31_					
	pH 7.4		pH 7.4		pH 5.5		pH 6.5		pH 7.4	
	MIC^b^	MBC^c^	MIC^b^	MBC^c^	MIC^b^	MBC^c^	MIC^b^	MBC^c^	MIC^b^	MBC^c^
1a	2	2	2	16	>200	>200	20	40	20	40
1b	1	1	2	4	>200	>200	15	20	15	20
2a	2	2	2	4	>200	>200	15	40	15	20
2b	1	>32	2	2	>200	>200	15	20	15	15
3	2	4	2	8	>200	>200	20	60	15	15
4	4	16	2	8	>200	>200	20	40	15	15
5	4	>32	16	32	>200	>200	25	40	15	20
6	1	>32	16	32	>200	>200	15	25	15	20
7	2	2	8	8	>200	>200	25	60	15	20
8	4	>32	2	4	>200	>200	20	30	15	15
9	2	2	4	4	>200	>200	15	20	15	15
10	2	2	2	4	>200	>200	20	40	15	15

^a^Colonies 4, 5 and 8 are vancomycin-intermediate *S*. *aureus* (VISA) based on the CLSI criteria (MIC = 4–8μg/mL) [28].

^b^Minimum inhibitory concentration (μg/mL).

^c^Minimum bactericidal concentration for 99.9% killing (μg/mL).

### *S*. *aureus* growth under acidic conditions

To evaluate the impact of pH and a type of acids on bacterial growth, *S*. *aureus* clinical isolates were grown in MH broth at three different pH values (5.5, 6.5 and 7.4). The broth (pH 7.4) was acidified using each one of three different acids (hydrochloric, acetic, and lactic acids) to give desired pH values. The initial bacterial suspension contained *S*. *aureus* with 2×10^6^ colony-forming units (CFUs) per ml. The bacterial suspensions with different pH values were incubated at 37°C for 24 hours. After the incubation, a 10-μl aliquot was removed from each well and inoculated onto agar plates for viable colony counting.

### Antibacterial susceptibility of *S*. *aureus* to PE_31_

The minimum inhibitory concentration (MIC) of the polymer was determined according the standard protocol for broth micro-dilution method in 96-well plates, published by the Clinical Laboratory and Standards Institute (CLSI) [18]. The bacterial isolates were grown to an early stationary phase in MH broth for twelve hours and were harvested via centrifugation (2500 g). The pH of MH broth (pH = 7.4) was adjusted to be 5.5 or 6.5 by adding lactic acid. The pH adjusted MH broth was inoculated with bacteria, and aliquots (150 μL) were transferred into a 96-well plate. The polymer was dissolved in MH broth with the pH adjusted to match the culture conditions and then serially diluted to give a range of polymer concentrations. The polymer stock solutions were added to the bacterial suspension on the plate. The final polymer concentrations in the plate ranged from 0.25 to 200 μg/mL. The plates with the bacterial suspension were placed in an EnSpire® multimode plate reader (Waltham, MA) and incubated at 37°C for 24 hours. The optical density of assay solution at 600 nm was measured every hour. The MIC value was defined as the lowest polymer concentration, in which no increase in the optical density was detected. The assay solutions were serially diluted and inoculated on agar plates, and the number of colonies was counted. Minimum bactericidal concentration (MBC) was determined as the polymer concentration for 99.9% reduction in the number of viable bacterial cells (colony forming unit, cfu) from the control without the polymer.

### Zeta potential measurement of bacteria

Overnight cultures of the bacteria grown at three different pHs (5.5, 6.5 and 7.4) were washed three times using 0.5 mM sodium phosphate buffer of the same pH. The washed bacteria were resuspended in the sodium phosphate buffer of the same pH to give a final OD_600_ of 0.4. A Malvern Zetasizer Nano ZS (Malvern Instruments, Worcestershire, England) was used to measure the zeta potential of bacterial samples. Potentials were calculated using the Smoluchowski equation for electrophoretic mobility at 25°C. For the calculation, the following parameters were used: 78.54 for a dielectric constant of the dispersant, 0.89 cP of viscosity, and 1.33 for refractive index. The zeta potential value was determined by 100 repetitions per sample. All measurements were performed in three independent experiments for each colony of *S*. *aureus*.

### Hemolysis assay

The hemolytic activity of the polymer was measured using an assay method as previously described [19]. The polymer was dissolved in phosphate buffered saline (PBS) at pH 5.5, 6.5, or 7.4, and the solutions were serially diluted in PBS of the same pH. The pH of PBS was adjusted by lactic acid. This series of polymer solutions were added to a solution of washed human red blood cells (RBCs). The final hematocrit was 5%, and the final polymer concentrations ranged from 8 to 1000 μg/ml. The assay plates were incubated at 37°C for 4 h with shaking (200 rpm). After the incubation, the plates were centrifuged at 3000 g for 5 min, and the supernatants were transferred to a new plate. The released hemoglobin was determined by measuring the absorbance of the supernatant at 576 nm. The hemolysis percentage was calculated by using the following formula:

$$\mathrm{Hemolysis}=\frac{{\mathrm{OD}}_{576}(\mathrm{Sample})-{\mathrm{OD}}_{576}(\mathrm{PBS})}{{\mathrm{OD}}_{576}(\mathrm{TritonX}100)-{\mathrm{OD}}_{576}(\mathrm{PBS})}\times 100$$ (1)

The negative control was PBS (pH 7.4) without polymer, and the positive control (100% lysis) was RBCs treated with 0.2% Triton X-100.

### Cytotoxicity of PE_31_ to human fibroblasts

Normal adult human dermal fibroblasts were used to evaluate the cytotoxic effects of PE_31_. A cell suspension (100 μL, density of 10^5^ cells/ml) in fibroblast basal medium was dispensed on 96-well tissue culture plates for 24 h and incubated at 37°C, under 5% CO_2_, to 80–90% confluence. The fibroblasts were treated with PE_31_ solution diluted in fibroblast basal medium adjusted to three different pH values (5.5, 6.5, or 7.4) at concentrations of 10, 50, 100 and 1000 μg/ml. The treated cells were incubated at 37°C for 24, 48 and 72 h. A CCK-8 solution (10 μL) was then added to each well. The plates were incubated for another two hours at 37°C, and the absorbance of solutions were measured at 450 nm. Cell viability (%) was determined as the relative value to that of cells in media pH 7.4 without the polymer, calculated using the following formula;

$$\mathrm{Cell}\_\mathrm{Viability}\_(\%)=\frac{{\mathrm{OD}}_{450}(\mathrm{Sample})-{\mathrm{OD}}_{450}(\mathrm{blank})}{{\mathrm{OD}}_{450}(\mathrm{Contol}\_\mathrm{pH}7.5)-{\mathrm{OD}}_{450}(\mathrm{blank})}\times 100$$ (2)

### Statistical analysis

All the experiments were carried out three times in duplicate samples. All analyses were performed using one- or two-way ANOVA when appropriate. The graphics were reported as the means and the standard deviation for the means. Statistical significance was defined p-values less than 0.05. All of the statistical procedures were performed using RStudio Version 0.98.932 (RStudio, Boston, MA).

## Results

### Antimicrobial polymer design and synthesis

**Fig 1:**
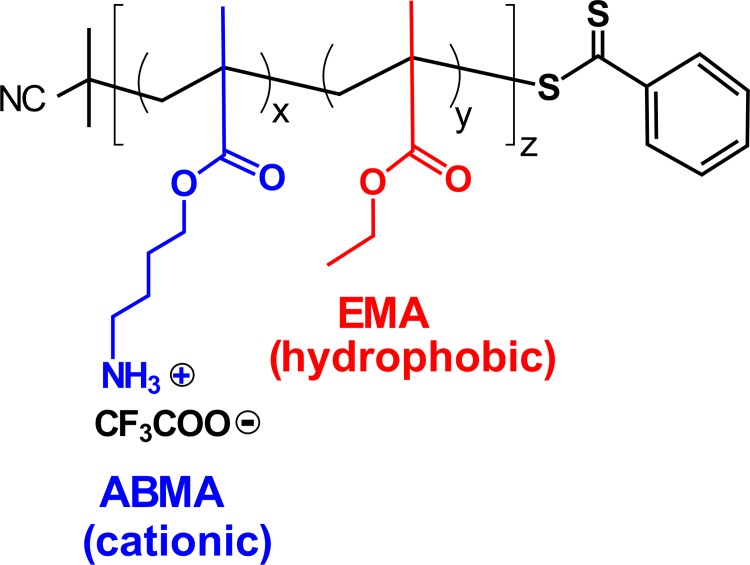
Chemical structure of random methacrylate copolymer PE_31_. The average mole percentage of EMA in a polymer chain was 31 mole %, and the degree of polymerization (DP) was 16. The mole percentage and DP were determined by ^1^H NMR analysis. The number average molecular weight (*M*_*n*_) of PE_31_ was 2,600 g/mol, which was calculated based on the DP and the molecular weights of monomers and chain transfer agents. The molecular weight of trifluoroacetic acid was excluded in the calculation of *M*_*n*_ for comparison with those of AMPs.

We previously designed, synthesized, and characterized a series of cationic, amphiphilic copolymers with antimicrobial activity [12]. We selected the methacrylate copolymer consisting of aminobutyl methacrylate (ABMA) and ethyl methacrylate (EMA) or poly(ABMA-EMA) (Fig 1) for further study because the polymer formulation showed both potent antimicrobial activity and a high degree of selectivity to bacteria over human cells [12]. Traditional antimicrobial polymers are high molecular weight polycations with quaternary ammonium groups modified with long alkyl groups [20–22]. By contrast, our polymer was designed to be relatively small and contain primary ammonium groups, which mimics the overall size and cationic moieties (from Lys residues) traditionally found in AMP sequences. The synthetic procedure of poly(ABMA-EMA) was previously reported [23]. The polymer was synthesized by RAFT polymerization (S1 Fig). While the resulting polymer composition could be altered by varying the ratio of monomers in the polymerization, we prepared the specific polymer as described below. This polymer is referred as PE_31_ through the report. The degree of polymerization (DP) of the PE_31_ was 16, yielding a number-average molecular weight of 2,600 g/mol, which comparable to low molecular weight α-helical AMPs such as the well-characterized natural AMP magainin 2 (2,467 g/mol) [24]. The mole percentage of EMA was 31 mole %, yielding an approximately 2:1 ratio of cationic to hydrophobic moieties in the average PE_31_ molecule (See S2 Fig for ^1^H NMR spectrum).

### pH- and acid-dependent bacterial growth

**Fig 2:**
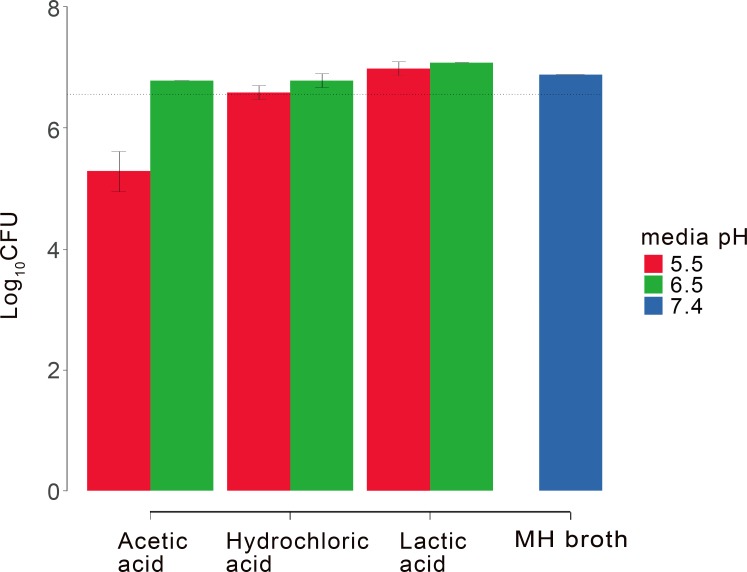
Effect of media pH and acids on *S*. *aureus* growth. 24-hour change in bacterial density of *S*.*aureus* grown at pH 5.5, 6.5, and 7.4 in MH broth. The pH of MH broth was adjusted by the acids indicated. The initial bacterial suspension contained *S*. *aureus* with 2×10^6^ colony-forming units (CFUs) per ml presented by a broken line. Results shown are the mean and standard deviation of three independent experiments in duplicate samples per condition.

Before examining the pH-dependent antimicrobial activity of PE_31_, we determined the general effect of pH on the growth of *S*. *aureus* as well as any influences from the type of acid used to modify solution pH (Fig 2). This is important to the interpretation of later results as any basal change in bacterial growth rates due to pH may affect the inhibitory effects of PE_31_. We selected lactic acid, acetic acid, and hydrochloric acid for this study. Lactic acid was included as it is one of acidic components in sweat from eccrine glands [17]. Acetic acid significantly decreased the bacterial growth at pH 5.5 (p < 0.01) as compared to pH 7.4 while no significant effect at pH 6.5. Lactic acid caused no significant reduction in the bacterial growth at pH 6.5 or 5.5 compared to the control. It is not clear at this point why lactic acid has no inhibitory effect on *S*. *aureus* unlike acetic acid. Based on these results, lactic acid was used to acidify buffer and media solutions in the following antibacterial assays.

### Antimicrobial activity of PE_31_ against MRSA clinical isolates

**Fig 3:**
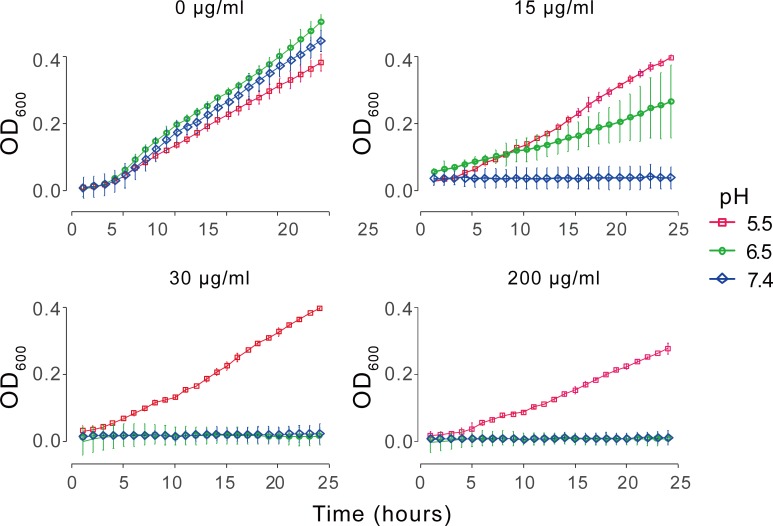
pH-dependent anti-staphylococcal activity of PE_31_. Representative growth curves of one strain of methicillin-resistant *S*. *aureus* (Strain No. 7) as measured by culture turbidity at 600 nm (OD_600_). PE_31_ concentration: 0 μg/mL, 15 μg/mL, 30 μg/mL, and 200 μg/mL. The data points and error bars represent mean and standard deviation of three replicates in duplicate samples per condition.

Next, we examined the antimicrobial activity of the PE_31_ polymer against MRSA clinical isolates. The minimum inhibitory concentration (MIC, concentration required to inhibit growth in an overnight culture) of vancomycin, mupirocin, and the polymer PE_31_ were determined by monitoring the turbidity (optical density) of bacterial cultures as a measure of bacterial growth (Fig 3) [18] The minimal bactericidal concentration of PE_31_ (MBC) was also determined as the concentration required to achieve 99.9% killing of bacteria. We included antibiotic mupirocin, which has been used to treat topical *S*. *aureus* infections [25] as a parallel for the long-range interest in using PE_31_ as a treatment of *S*. *aureus* skin infections. As anticipated from previous reports, vancomycin and mupirocin inhibited the growth of MRSA with MIC and MBC values of 2–32 μg/mL at pH 7.4, depending on the individual strain [26, 27]. Among the tested MRSA clinical isolates, the MIC values of colonies No. 4, 5, and 8 are 4 μg/mL, which are greater than the MIC values of 1–2 μg/mL for other colonies. These strains are classified as vancomycin-intermediate *S*. *aureus* (VISA), according to the criteria (MIC = 4–8 μg/mL) published by the Clinical and Laboratory Standards Institute (CLSI) [28][ref] The polymer PE_31_ inhibited growth of MRSA strains at pH 7.4 with MIC values of 15 or 20 μg/mL against all strains tested (Table 1). The PE_31_ yielded MBC values which were the same or close to the MIC values, suggesting that PE_31_ exerts the inhibitory effects by killing *S*. *aureus*, i.e., a completely bactericidal mechanism.

### pH-dependent anti-*S*. *aureus* activity of PE_31_

The pH responsiveness of the anti-staphylococcal activity of PE_31_ was determined. We used three different pH conditions: neutral (pH = 7.4) and low pH conditions (pH = 6.5 and 5.5), which reflect the infected (exposure of pH-neutral subcutaneous tissue) and healthy skin conditions, respectively. Inhibition of bacterial growth was found to be both dose-dependent and pH-dependent, with PE_31_ being highly effective at pH 7.3 but exhibiting no detectable antimicrobial effect at pH 5.5 (Table 1) (Fig 3). The MIC values were 15 or 20 μg/mL at pH 7.4, and the MIC values were parallel or slightly increased at pH 6.5 to 15–60 μg/mL (Table 1). At pH 5.5, all MIC values were greater than 200 μg/mL, indicating significant loss of function. The MBC values also increased from 15 or 20 μg/mL at pH 7.4 to > 200 μg/mL at pH 5.5. Together, these results indicate that PE_31_ is no longer active against *S*. *aureus* under acidic conditions and exhibits clear pH-dependent activity.

**Fig 4:**
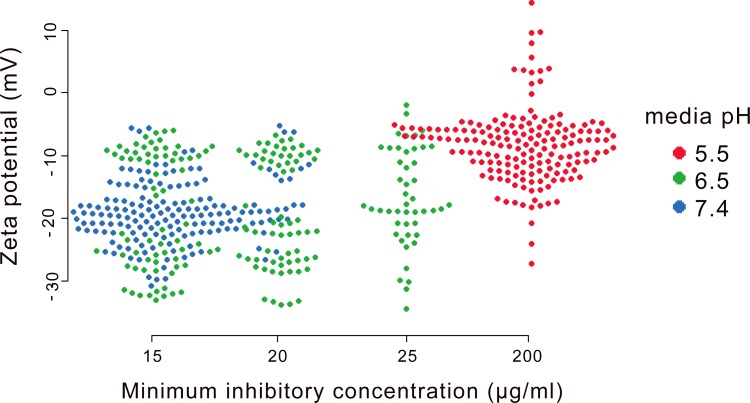
Relationship between pH, bacterial zeta potential, and minimum inhibitory concentration of PE_31_. Data reflect multiple replicate zeta-potential measurements and MIC measurements across ten clinical blood isolates of methicillin-resistant *S*. *aureus*.

Considering the primary binding interaction of PE_31_ to the bacterial cell surface is driven by coulombic interactions, binding could be affected if the charge on the *S*. *aureus* cell surface changes when the pH of the growth medium changes. If the net surface charge decreases under acidic conditions, the electrostatic binding of PE_31_ to bacteria would be reduced, resulting in decreased activity. To test this hypothesis, we determined the zeta potentials of all strains studied at various pH conditions. As shown in Fig 4, the zeta potential of *S*. *aureus* is strongly correlated to the culture media pH values. The bacteria at the pH 7.4 were found in more negative zeta potential, while most bacteria in pH 5.5 have less negative potential (p < 0.05). The result indicates that the bacterial surface of *S*. *aureus* is more negatively charged at pH 7.4 than under acidic conditions. Similar pH-dependent effects on the zeta potential of *S*. *aureus* have been also reported in literature [29, 30].

The MIC values of PE_31_ were also correlated to the pH of the bacterial growth media (Table 1 and Fig 4). The MIC values were lower for higher pH values, indicating that PE_31_ was more active against *S*. *aureus* with a more negatively charged surface. The surface of the bacterium also becomes more negatively charged as pH was increased, which would enhance the electrostatic binding of PE_31_ the *S*. *aureus* cell surface. However, as discussed below, the overall hydrophobicity of PE_31_ could be also increased as the solution pH was increased, which may enhance the membrane permeability. Taken together, the pH-dependent activity of this polymer may result from a combination of enhancing both hydrophobic and electrostatic components of the mechanism of action in binding and membrane disruption.

### Cytotoxicity of PE_31_ to human cells

**Fig 5:**
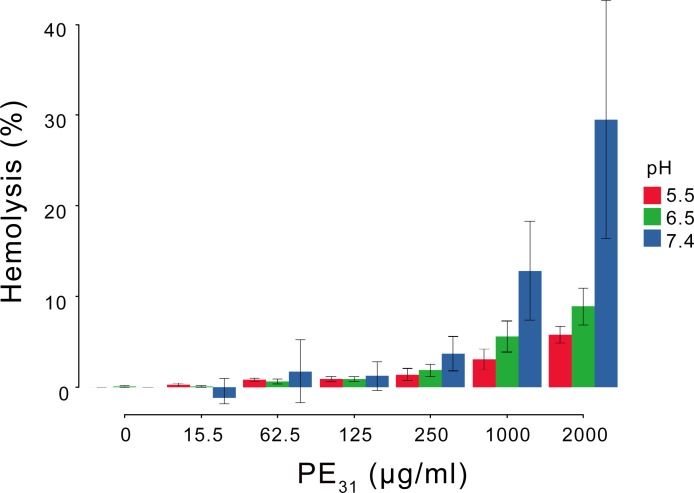
Hemolytic activity of PE_31_ against RBCs. Reported values are mean and standard deviation of three replicates in duplicate samples.

The *in vitro* cytotoxicity of PE_31_ to host cells was examined using several approaches. First, a standard hemolysis assay was performed to determine if PE_31_ disrupts human red blood cell (RBC) membranes. If PE_31_ disrupts the RBC membranes, hemoglobin is released into the solution. In this assay, washed human RBCs were incubated with varied concentrations of PE_31_ at the three pH values previously tested (pH 5.5, 6.6, and 7.4). The hemolytic activity of PE_31_ was measured as the percentage of hemoglobin release from RBCs normalized by a detergent control to induce complete RBC lysis. In general, the percent hemolysis induced by PE_31_ increased as PE_31_ concentration increased. Notably while hemolysis was modest at the highest concentrations tested, (percent hemolysis ∼ 30% at pH 7.4 at 2000 μg/mL), there was negligible hemolytic activity until the concentrations of polymer were well above the MBC value (15–40 μg/mL at pH 7.4) (Fig 5). The hemolytic activity at pH 7.4 was higher than that in either of the more acidic conditions, indicating that the hemolytic activity of PE_31_ was increased as the pH was increased, which is consistent with the pH-dependent anti-*S*. *aureus* activity.

**Fig 6:**
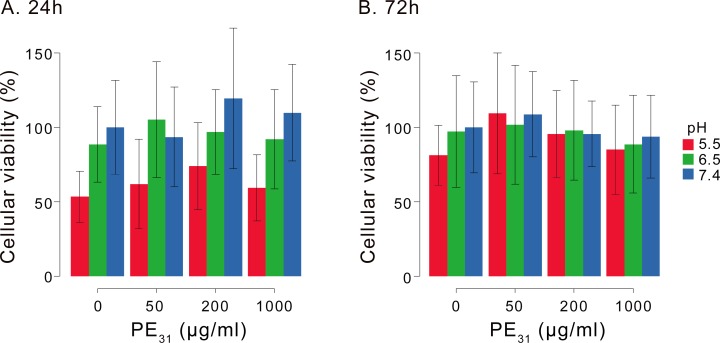
**The cytotoxicity of PE**_**31**_ **against cultured human dermal fibroblasts after incubation for 24 hours (A) or 72 hours (B).** The cell viability was determined as the relative value to that of cells at pH 7.4 without PE_31_. Reported values represent the mean and standard deviation of three independent experiments in duplicate samples per condition.

Next, in an attempt to more accurately reflect the intended application conditions of this molecule, the cytotoxicity of PE_31_ against human dermal fibroblasts was studied. The cell viability of fibroblasts was determined after incubation with PE_31_ for 24 or 72 hours. The cell viability (%) was determined relative to that of fibroblasts at pH 7.4 in the absence of PE_31_. It is important to highlight that after 24 hours, the viability of fibroblasts at pH 5.5 without PE_31_ is lower than that of fibroblasts at pH 7.4, regardless of polymer concentration, indicating that the acidic conditions potentially reduced the metabolic activity of cells and/or cell proliferation. When the cell viability data at the same pH values (pH 5.5–7.4) were compared, there were no significant differences in the viability after incubation with PE_31_ for 24 or 72 hours (Fig 6). After 72 hours, it appears that the effect of pH on the cell viability is no longer as severe, and the average viability is > 80% up to 1000 μg/mL PE_31_. The range of polymer concentration tested in this study (50–1000 μg/mL) is well above the MIC values of polymer at pH 6.5 and 7.4 (MIC = 15–60 μg/mL). The results from the hemolysis and cytotoxicity assays indicate that PE_31_ was selective to *S*. *aureus* over both human RBCs and human dermal fibroblasts and appeared to exhibit an easily accessible therapeutic window for this application.

## Discussion

The results from this study show that the cationic amphiphilic random copolymer PE_31_ acts a pH- and dose-dependent antimicrobial agent. PE_31_ inhibited bacterial growth of clinically isolated drug-resistant *S*. *aureus* (MRSA and VISA) at neutral pH 7.4. Most of the MBC and MIC values of PE_31_ against the same bacterial isolates were the same or close (within 3 fold) (Table 1), indicating that the polymer exerted antimicrobial effects against MRSA and VISA via a bactericidal mechanism. However, PE_31_ was effectively inactive at pH 5.5, the approximate conditions similar to the pH environment of human skin. The bactericidal activity of this polymer would favor eradication of *S*. *aureus* from dermal infection sites. In addition, PE_31_ was active with similar MIC value against VISA strains and other vancomycin susceptible MRSA strains. These results suggest that the antimicrobial mechanism of PE_31_ was not related to the standard antibiotic resistance mechanisms of *S*. *aureus*, consistent with the proposed membrane disrupting mechanism of PE_31_. While the data indicating pH dependent activity is clear, the underlying mechanism for the change in activity is still a ripe area for investigation. It is possible that the sharp differences in activity stem from pH-dependent changes in the polymer itself and/or the target bacterium.

There are several possibilities for pH dependent changes in the bacterial physiology in response to pH. The zeta-potential experiments (Fig 4) show that the overall surface charge density on the *S*.*aureus* cell membrane changes in response to pH. However, it is not clear *why* the surface charge of *S*. *aureus* is pH dependent. The increased negative surface charge of *S*. *aureus* could be attributed to acidic groups of biopolymers such as teichoic acids, major components of *S*. *aureus* cell wall. However, the pKa of phosphate groups of teichoic acid is ~2, suggesting that the teichoic acid is fully anionic across the pH range tested here (pH 5.5 to 7.4). We speculate that other acidic or basic groups in the *S*. *aureus* cell wall could contribute to the pH-dependent differences, although no specific groups have been identified in the literature.

Changes in *S*. *aureus* gene expression response to environmental pH may also contribute to the pH-dependent activity of PE_31_. The GraRS two-component sensor system in *S*. *aureus* has previously been shown to respond to cationic, amphiphilic hydrophobic peptides and polymers [31–35]. Deletion of this two-component sensor has been shown to modulate the susceptibility of *S*. *aureus* in a pH dependent manner. Additionally, studies on GraRS mediated responses to host defense peptides showed similar pH dependent activity with several peptides exhibiting lower anti-*Staphylococcal* activity at low pH. This regulatory sensor controls the expression of several genes (*mprF* and *dlt*) which modulate cell surface charges in *S*. *aureus*. Other staphylococcal regulatory pathways have also been linked to cationic, amphiphilic peptides and polymers include the NsaSR system involved in sensing cell envelope stress [35, 36] and the SaeRS regulatory system involved in virulence, which was also shown to be influenced by the pH of the medium [37]. Signaling through these bacterial sensors are clearly linked to pH dependent activity of antimicrobial peptides, thus they may also be involved in the pH dependent activity of the cationic, amphiphilic PE_31_.

Due to the low pH conditions PE_31_ was exposed to for 24 h in the assay and the reactivity of the ester groups in the polymer side chains, hydrolysis of the esters in the polymer side chains could potentially contribute to the loss of function. The stability of PE_31_ under acidic conditions was analyzed by incubating at pH 5.5 followed by examination of the polymer structure by ^1^H NMR (S3 Fig and S1 Text). The results indicated that there was no significant change in the mole ratio of cationic side chains and ethyl side chains before and after the incubation at 37°C for 24 hours in 0.1M sodium acetate/acetic acid buffer of pH 5.5. While lactic acid was used for the antibacterial assay, the signal from lactic acid in the ^1^H NMR spectrum overlapped with those from the polymer side chains. Therefore, we could not determine the effect of lactic acid on the hydrolysis of side chain esters. While the acidic conditions did not significantly affect the ratio of hydrophobic and cationic moieties of the polymer, the NMR data is inconclusive regarding any breakdown of the esters and resulting liberation of side chains. Any potential could be a contributing factor in the loss of activity at lower pH. However, any hydrolysis of the polymer side chains that impacts overall efficacy would need to be extremely rapid to be consistent with our data. In Fig 3, the untreated *S*. *aureus* at pH 5.5 grew with a similar profile to all of the pH 5.5 samples treated with PE_31_, even at the highest concentration (200mg/ml, ~10x greater than the MIC at pH 6.5). If polymer degradation were a significant factor in loss of activity, there would undoubtedly be some fraction of in-tact polymer at the early time points to inhibit growth. Additionally, since the PE_31_ was shown to be bactericidal, removal of the PE_31_ via hydrolysis would not allow for a rapid recovery of the *S*. *aureus* exposed to the in-tact polymers, resulting in a delay or shift in the growth curves, which was not observed. While future studies on the breakdown hydrolysis of the polymer side chain esters under different conditions are warranted, it does not appear that this is the primary factor affecting pH-dependent activity. Our results suggest that other factors could also contribute to the pH dependent activity of PE_31_ against *S*. *aureus* as discussed below.

The pH-dependent activity of PE_31_ against *S*. *aureus* might also be attributed to the changes in the amphiphilic properties of the polymer chain. The cationic groups of PE_31_ are expected to bind to the anionic bacterial membranes through electrostatic interactions, leading to selective activity to bacteria over human cells. Upon binding to bacterial membranes, the hydrophobic side chains of PE_31_ are inserted into the hydrophobic region of the bacterial lipid membrane, which subsequently causes membrane disruption and ultimately bacterial cell death. However, if the polymer chain is highly hydrophobic, the hydrophobic binding would be dominant, resulting in non-specific binding to bacteria and human cells. Therefore, the balance of cationic and hydrophobic characteristics is the key determinant in the design of cationic amphiphilic polymers with potent activity and cell selectivity [12]. Accordingly, changes in the cationic-hydrophobic balance, caused by changing the solution pH, could alter the antimicrobial activity of PE_31_. PE_31_ showed increased hemolytic activity at pH 7.4 against human RBCs, which have primarily zwitterionic cell membrane surface. The result may indicate an increase in the net hydrophobicity of PE_31_ at pH 7.4. Any increase in net hydrophobicity of PE_31_ is likely to increase the ability of PE_31_ to disrupt bacterial membranes, resulting in the higher antimicrobial activity. Alternatively, the behavior of the polymer itself may be influenced by the environmental pH. Amphiphilic random copolymers have been known to form intramolecular or intermolecular compact, micelle-like aggregates due to the association of hydrophobic side chains in water [38–40]. Similarly, the polymer chains of PE_31_ do not adopt regular, folded, three-dimensional structures in solution, however they are also likely to exist in such a compact or “collapsed” state in solution. This collapsed state is conceptually similar to the folding of proteins with hydrophobic groups sequestered at the interior of the collapsed polymer and hydrophilic and charged groups orienting toward the surface. Our previous work indicated that the polymer would likely contain an equal fraction of charged side chains at both pH 5.5 and 7.4 [13]. However, it is possible that the overall shape or dynamic structure of the collapsed chain could be affected by the pH. This could result in differential exposure of hydrophobic groups, resulting in the observed pH-dependent activity. Additionally, the solution aggregation state of the polymer may also be influenced by the pH. Changes in polymer aggregation in solution could affect the binding of the polymer by shifting the thermodynamic equilibrium between the solution/unbound and the membrane-associated states. While we currently have no direct evidence regarding the polymer solution properties, the aggregation and collapsed states of polymer chains in the assay media are important factors to be investigated to determine the underlying causes of the pH-dependent activity of PE_31_.

We envision that a future clinical application of an antimicrobial polymer as a topical agent for treatment of infection on skin or subcutaneous tissues. The data clearly showed PE_31_ was active against MRSA at neutral pH but inactive at the low pH 5.5, reflective of infected and healthy skin tissue, respectively. It is generally accepted that bacterial infection sites are acidic owing to the abscess formation through the normal host defense response [41]. Accordingly, previous studies in the literature were conducted for antimicrobial polymers or drug delivery systems with acid-activated mechanisms that were intended to target the acidic infection sites [42, 43]. Our study reported here appears to contradict with such traditional approaches using acid-activation mechanisms. However, the normal skin surface is acidic due to the acid mantle to prevent growth of pathogenic bacteria, while the pH of infectious sites become more neutral because of the lack of stratum corneum function maintaining acidic skin surface pH [44]. In this case, the reported models of acid-activation of antimicrobials would be ineffective due to the compounds becoming active under “normal” conditions and less active or inactive at the infection site. Therefore, the strategy to develop an antimicrobial which is active at pH-neutral infection sites, but inactive at the acidic health skin surfaces can specifically be applied to these conditions. Additionally, our strategy of acid-inactivated antimicrobial polymers may also minimize undesired side effects associated with non-specific killing of commensal bacteria, adapted to the normal acidic skin environment. However, while we propose this new strategy, this will require significant future studies including *in vivo* testing to confirm the efficacy and retention of the pH-sensitive activity of PE_31_ in a true skin-infection model, as well as a thorough mechanistic analysis of *in vitro* and *in vivo* polymer degradation via hydrolysis of side chain esters.

## Conclusion

In summary, cationic amphiphilic copolymer PE_31_ showed bactericidal activity against drug-resistant *S*. *aureus* (MRSA and VISA) clinical isolates. PE_31_ was active against *S*. *aureus* at neutral pH 7.3, but inactive at pH 5.5. PE_31_ did not cause any significant hemolytic activity to human RBCs or cytotoxicity to human dermal fibroblasts. Our work suggests potential future utility of PE_31_ as a selective agent with inherent pH-responsive activity against drug-resistant *S*. *aureus*. We propose a new potential strategy using the pH-responsive polymer, which would target pH neutral *S*. *aureus* in skin infection sites, but it is inactive at the acidic health skin. However, we acknowledge that *in vivo* efficacy of the polymer needs to be thoroughly investigated for further development of PE_31_ as a topical antimicrobial agent.

## Supporting Information

S1 Fig Synthetic scheme of PE_31_. (PDF)

S2 Fig ^1^H NMR spectrum of PE_31_. (PDF)

S3 Fig ^1^H NMR spectrum of PE_31_ after incubation at 37°C in 0.1 M acetic buffer of pH 5.5 for 24 hours. (PDF)

S1 Table Characterization of boc-protected and de-protected PE_31_. (PDF)

S1 Text Stability of PE31 under an acidic condition. (PDF)
