# Supplementary material for: A Cationic Amphiphilic Random Copolymer with pH-Responsive Activity against Methicillin-Resistant Staphylococcus aureus
Source: PLoS One. 2017 Jan 6;12(1):e0169262. doi: 10.1371/journal.pone.0169262 (PMC5217864; doi:10.1371/journal.pone.0169262)
Supplement: S1 Fig — (PDF) [file pone.0169262.s001.pdf]

A Cationic Amphiphilic Random Copolymer with pH-Responsive Activity against Methicillin-Resistant *Staphylococcus aureus*

Sungyoup Hong, Haruko Takahashi, Enrico T. Nadres, Hamid Mortazavian,

Gregory A. Caputo, John G. Younger, Kenichi Kuroda

S1 Fig. Synthetic scheme of PE_{31}.

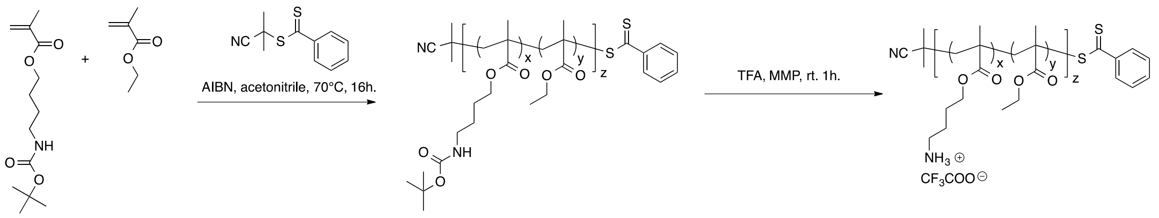

The monomer 4-((tert-butoxycarbonyl)amino) butyl methacrylate (ABMA) was synthesized according to the previous report (Fig S1) [1]. ABMA (7 mmol, 3.5 mL of 2 M in acetonitrile), ethyl methacrylate (EMA) monomer (3 mmol, 1.5 mL of 2 M in acetonitrile), 2-cyanoprop-2-yl-dithiobenzoate chain transfer agent (1 mmol, 221 mg) and AIBN radical initiator (0.1 mmol, 16 mg) (Fig. S1) [2]. The mixture was flushed with nitrogen for 5 min and stirred at 70ºC. After 16 hours, the polymerization was stopped by cooling the reaction vial in a dry ice/acetone bath. The solvent was removed under reduced pressure, and the residue was dissolved in dichloromethane. The solution was poured into excess hexanes to isolate the polymer product as a precipitate. This precipitation process was repeated twice. The degree of polymerization (DP) was determined by ^{1}H NMR spectroscopy by comparing the integrated peaks of benzyl group of chain transfer agent at the polymer ω-end and side chains. The molecular weight of polymer was determined by GPC (eluent THF, polystyrene standard, refractive index detector): Mn = 2774, Mw = 3117, PDI=1.12 (RI).

De-protection and characterization of PE_{31}

The Boc-protected polymer (1.00 g) was first mixed with methyl 3-mercaptopropionate (0.5 mL) and then dissolved in TFA (5 mL). After stirring for 30 min, TFA was removed by blowing with nitrogen gas. The residue was dissolved in methanol and precipitated in excess diethyl ether. The precipitated polymer was dissolved in distilled water and lyophilized, yielding a light pink fluffy product (0.9391g). 1H NMR (CD3OD, 400 MHz) δ 8.18-7.35 (m), 4.25-3.83 (m), 3.09-2.84 (m), 2.16-1.82 (m), 1.80-1.65 (m), 1.46-1.17 (m), 1.13-0.80 (m) (Fig S2). The degree of polymerization (DP) was determined by comparing integrated peaks of benzyl group of chain transfer agent at the polymer ω-end (signal d) and side chains (signal b + c) in the 1H NMR spectra (Fig S1) The mole percentage of EMA was determined by comparing integrated peaks of butylene groups of Boc-ABMA and ethylene groups of EMA in the 1H NMR spectra (signals a and b + c). The DP of the deprotected polymer was 15.9, and mole percentage of EMA was 30.6 mole %.
