# Supplementary material for: A Cationic Amphiphilic Random Copolymer with pH-Responsive Activity against Methicillin-Resistant Staphylococcus aureus
Source: PLoS One. 2017 Jan 6;12(1):e0169262. doi: 10.1371/journal.pone.0169262 (PMC5217864; doi:10.1371/journal.pone.0169262)

A Cationic Amphiphilic Random Copolymer with pH-Responsive Activity against Methicillin-Resistant *Staphylococcus aureus*

Sungyoup Hong, Haruko Takahashi, Enrico T. Nadres, Hamid Mortazavian,

Gregory A. Caputo, John G. Younger, Kenichi Kuroda

S3 Fig. ^{1}H NMR spectrum of PE_{31} after incubation at 37°C in 0.1M acetic buffer of pH 5.5 for 24 hours.

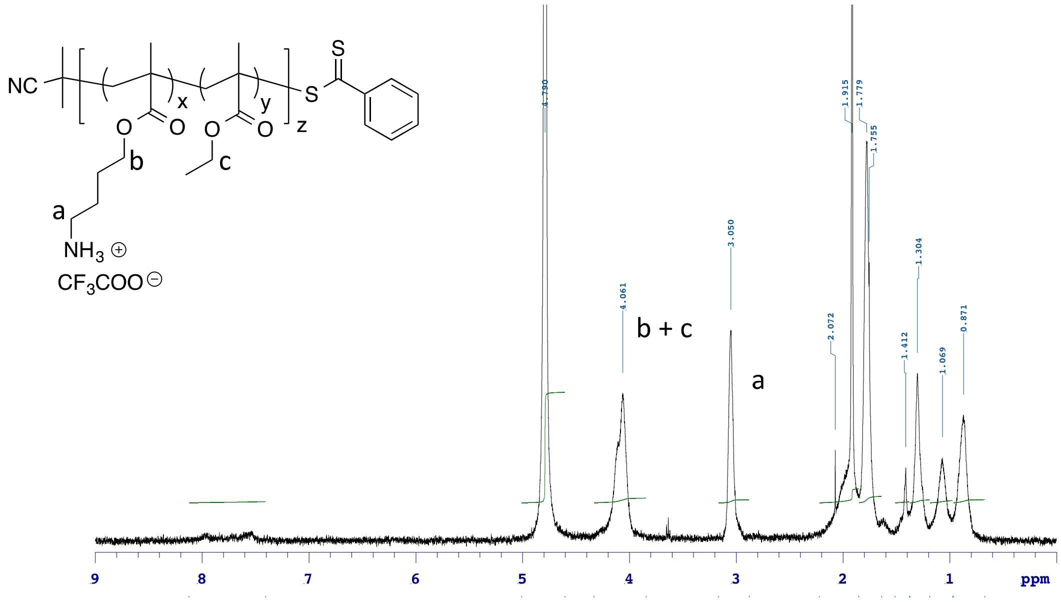

Supplement: S3 Fig — (PDF) [file pone.0169262.s003.pdf]
