# Supplementary material for: A Cationic Amphiphilic Random Copolymer with pH-Responsive Activity against Methicillin-Resistant Staphylococcus aureus
Source: PLoS One. 2017 Jan 6;12(1):e0169262. doi: 10.1371/journal.pone.0169262 (PMC5217864; doi:10.1371/journal.pone.0169262)
Supplement: S1 Table — (PDF) [file pone.0169262.s004.pdf]

A Cationic Amphiphilic Random Copolymer with pH-Responsive Activity against Methicillin-Resistant *Staphylococcus aureus*

Sungyoup Hong, Haruko Takahashi, Enrico T. Nadres, Hamid Mortazavian,

Gregory A. Caputo, John G. Younger, Kenichi Kuroda

S1 Table. Characterization of boc-protected and de-protected PE_{31}.

**Boc-protected PE_{31}**				**De-protected PE_{31}**		
M_n [a] (NMR)	M_w/M_n [b] (GPC)	DP [c]	EMA [d] (mole %)	M_n [a] (NMR)	DP [c]	EMA [d] (mole %)
3430	1.12	15.0	30.3	3770 (w/ TFA)<br>2550 (w/o TFA)	15.9	30.6

[a] Calculated based on DP and molecular weights of monomer units and chain transfer agent.

[b] Determined by GPC in THF, polystyrene calibration, refractive index detector.

[c] Calculated by comparing integrated peaks of benzyl group of chain transfer agent at the polymer ω-end and side chains in the ^{1}H NMR spectra.

[d] Mole % of EMA relative to the total number of monomers in a polymer chain.
