# Supplementary material for: A Cationic Amphiphilic Random Copolymer with pH-Responsive Activity against Methicillin-Resistant Staphylococcus aureus
Source: PLoS One. 2017 Jan 6;12(1):e0169262. doi: 10.1371/journal.pone.0169262 (PMC5217864; doi:10.1371/journal.pone.0169262)
Supplement: S1 Text — (PDF) [file pone.0169262.s005.pdf]

A Cationic Amphiphilic Random Copolymer with pH-Responsive Activity against Methicillin-Resistant *Staphylococcus aureus*

Sungyoup Hong, Haruko Takahashi, Enrico T. Nadres, Hamid Mortazavian, Gregory A. Caputo, John G. Younger, Kenichi Kuroda

S1 text. Stability of PE_{31} under an acidic condition

Experimental. The stability of ester groups of PE_{31} under acidic conditions was investigated using acetic acid buffer of pH 5.5. A solution of PE_{31} in 0.1M acetic acid buffer (10 mg/ml, 1 mL) was incubated at 37 °C for 24 h. The solvent of polymer solution was removed under reduced pressure at room temperature. The resultant residue was dissolved in deutrated water (D_2O) for ^{1}H NMR analysis. We also attempted the same experiment using lactic acid because the pH of MH broth for the bacterial assays was adjusted by lactic acid. However, residual lactic acid showed a signal at 4.0 ppm in the ^{1}H NMR spectrum (not shown), which overlapped with the singles from the polymer side chains and did not allow determining the changes in the side chains due to hydrolysis of the ester groups. Therefore, we used acetic acid to prepare acidic conditions but not lactic acid.

Results. The signal from the benzyl group at the end of polymer chain was not distinctive. Therefore, the degree of polymerization (DP) could not be determined. The mole percentage of ABMA and EMA was determined by comparing integrated peaks of butylene groups of ABMA and ethylene groups of EMA in the ^{1}H NMR spectrum (signals a and b + c). The mole percentage of ABMA and EMA after incubation at pH 5.5 for 24 hours was 66 mol.% and 34 mol.%, respectively. The mole percentages of monomers did not change significantly

from the initial values (70 mol. % for ABMA and 30 mol.% for EMA) before the exposure to acid.
